# Imaging the DNA damage response with PET and SPECT

**DOI:** 10.1007/s00259-016-3604-1

**Published:** 2017-01-05

**Authors:** James C. Knight, Sofia Koustoulidou, Bart Cornelissen

**Affiliations:** CR-UK/MRC Oxford Institute for Radiation Oncology, Department of Oncology, University of Oxford, Old Road Campus Research Building, Off Roosevelt Drive, Oxford, OX3 7LJ UK

**Keywords:** DNA damage, PET, SPECT, Molecular Imaging, γH2AX, PARP

## Abstract

DNA integrity is constantly challenged by endogenous and exogenous factors that can alter the DNA sequence, leading to mutagenesis, aberrant transcriptional activity, and cytotoxicity. Left unrepaired, damaged DNA can ultimately lead to the development of cancer. To overcome this threat, a series of complex mechanisms collectively known as the DNA damage response (DDR) are able to detect the various types of DNA damage that can occur and stimulate the appropriate repair process. Each DNA damage repair pathway leads to the recruitment, upregulation, or activation of specific proteins within the nucleus, which, in some cases, can represent attractive targets for molecular imaging. Given the well-established involvement of DDR during tumorigenesis and cancer therapy, the ability to monitor these repair processes non-invasively using nuclear imaging techniques may facilitate the earlier detection of cancer and may also assist in monitoring response to DNA damaging treatment. This review article aims to provide an overview of recent efforts to develop PET and SPECT radiotracers for imaging of DNA damage repair proteins.

## Introduction

The DNA double helix within every cell of the human body is constantly exposed to damaging agents, and, consequently, tens of thousands of DNA lesions occur per cell each day [1]. If a lesion is not correctly repaired, it may cause the cell to become senescent, apoptotic, or even malignant. Over the last few decades, a multitude of endogenous and exogenous causes of DNA damage have been identified [2, 3]. Endogenous processes are responsible for the vast majority of DNA damage and can be divided into three main categories: oxidative (i.e. produced by reactive oxygen species), hydrolytic (e.g. deamination of cytosine to uracil), and alkylation reactions (e.g. methylation of the N7-position of guanine residues) [4]. As exogenous sources of DNA damage, ultraviolet light and ionizing radiation have been found to be among the most prevailing factors, causing single-strand breaks (SSBs) and double-strand breaks (DSBs), respectively [5]. In addition, certain chemotherapy drugs used for cancer treatment, industrial chemicals, and carcinogens associated with tobacco products are well recognised to cause DNA damage.

**Fig. 1:**
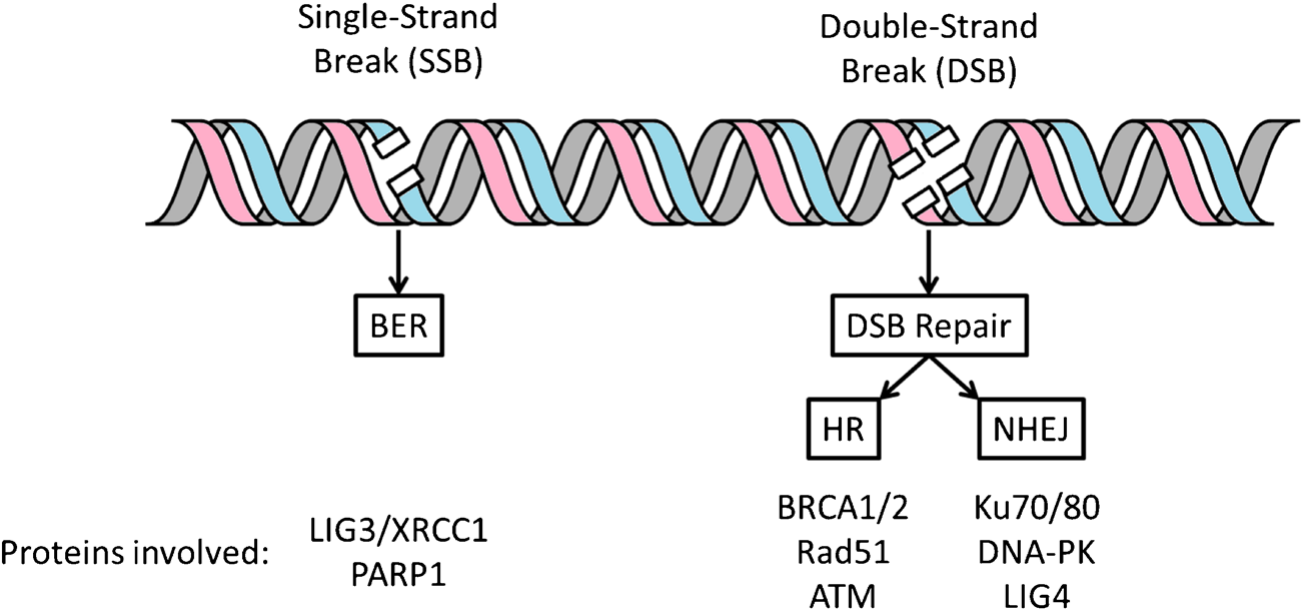
A simplified overview of the DNA damage response and the main targets involved. BER, Base Excision Repair; HR, Homologous Recombination; NHEJ, Non-Homologous End Joining; LIG3, DNA Ligase 3; XRCC1, X-ray repair cross-complementing protein 1; PARP-1, poly (ADP-ribose) polymerase 1; BRCA1/2, Breast Cancer 1/2; ATM, Ataxia Telangiectasia Mutated; DNA-PK, DNA-dependent protein kinase catalytic subunit

The recognition and repair of DNA damage is achieved by a set of complex yet finely tuned DNA damage response (DDR) signalling pathways that inhibit cell cycle progression and repair DNA lesions by a variety of mechanisms (Fig. 1). The excellent level of control over these processes ultimately minimises genomic instability and impedes tumorigenesis [6]. Defects in this defensive mechanism have been found to occur with significantly higher prevalence in many human cancers compared to normal tissues [7–11]. As a consequence, extensive DNA damage and DDR signalling is present and critically important in virtually all stages of tumour development, from dysplasia to advanced metastatic disease [12, 13].

Therefore, the ability to monitor DDR in vivo in a non-invasive manner via molecular imaging is an attractive prospect as the information obtained from these techniques could facilitate the earlier detection of several cancer types. Furthermore, as most cancer therapies are designed to cause DNA damage, these techniques may also provide a rapid and broadly applicable means of evaluating response to therapy. In this review article, we discuss potentially valuable biomarkers, which arise during the major cellular responses to SSBs and DSBs, and we also evaluate recent efforts to monitor these biomarkers non-invasively in vivo using PET and SPECT radiotracers.

## Single-strand break repair mechanisms

**Fig. 2:**
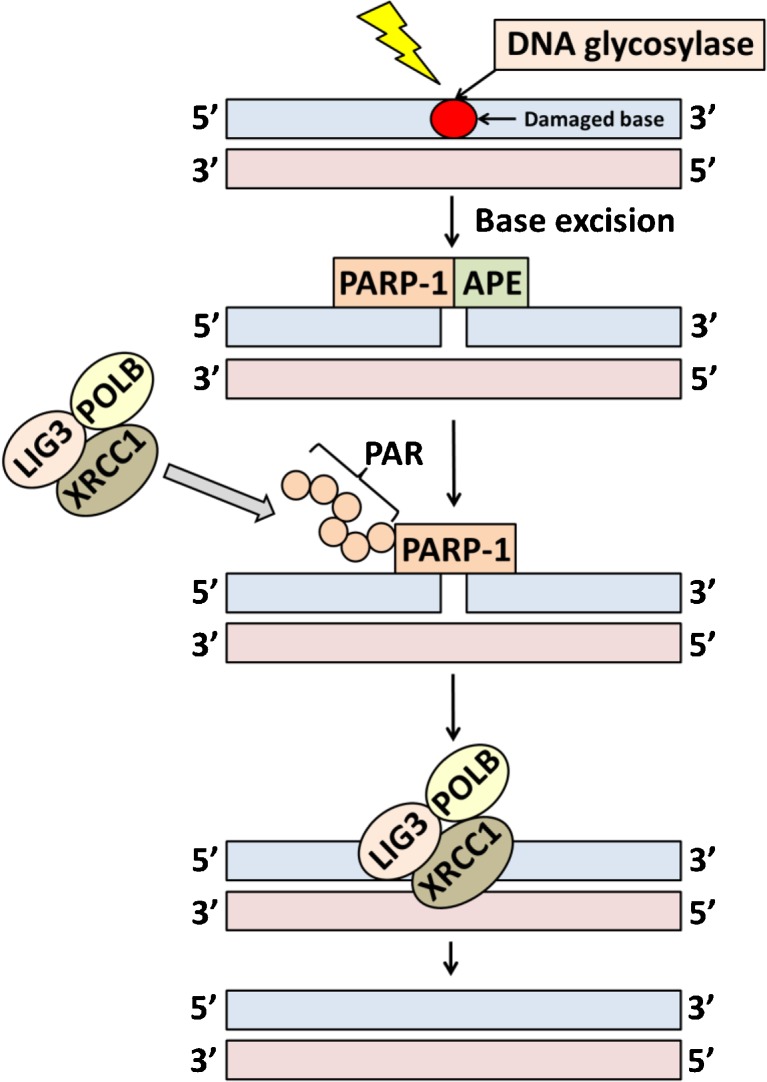
A simplified diagram showing the major steps in short-patch base excision repair pathway. In the presence of DNA damage, PARP-1 is activated upon binding to SSBs, leading to recruitment of BER proteins. These proteins will then identify and repair the damage

The repair of SSBs in DNA is mainly facilitated by base excision repair (BER) [14]. Deficiencies and mutations of proteins in this pathway are linked to genomic instability, aging, and cancer [15]. BER has two sub-pathways referred to as short-patch repair (Fig. 2) and long-patch repair; the former being responsible for up to 90% of all BER [16]. In brief, short-patch BER is based on five major steps: (*i*) recognition of the damaged base by a DNA glycosylase and the consequent removal of the base, creating an apurinic or apyrimidinic (AP) site intermediate, (*ii*) incision of the abasic site by an AP endonuclease (APE) or AP lyase, (*iii*) removal of the remaining sugar fragment by a lyase or phosphodiesterase, (*iv*) filling of the remaining gap by a DNA polymerase (commonly, DNA polymerase β, POLB) with the correct nucleotide, and finally (*v*) sealing of the remaining nick by a DNA ligase (LIG1 or LIG3/XRCC1 complex) [17].

Among the many sensors involved in the BER repair of SSBs, poly(ADP-ribose) polymerase 1 (PARP-1) is particularly influential in this process [18–21]. Upon binding to nicked DNA, PARP-1 cleaves nicotinamide adenine dinucleotide (NADb), whereupon it catalyses the polymerisation of ADP-ribose units into long, branched chains of poly(ADP-ribose) (PAR) [22]. While the chief target for poly(ADP-ribosylation) is PARP-1 itself via automodification, other DNA damage repair proteins and histones are also PARylated. PAR is thereafter responsible for the recruitment of additional DDR proteins which cooperate for the completion of SSB repair [23–25]. Small molecule inhibitors of PARP-1 have the ability to disrupt the BER repair pathway, leading to collapsed replication forks and ultimately DSBs upon replication [26–30]. Several key studies have since shown that normal cells can compensate in these circumstances by relying on other repair mechanisms, such as homologous recombination (HR), which act to restore the original DNA sequence [31]. In accordance with these observations, studies focused on the genetic removal of PARP-1 have found no significant effect upon the frequency of tumour development [31]. Notably, however, cancer cells with defects in HR (most commonly arising from mutations of BRCA1 and BRCA2 proteins) have exhibited vastly increased sensitivity to PARP-1 inhibitors [32, 33]. In such cases, damaged DNA either persists unrepaired or is subjected to a more error-prone DNA repair mechanism (e.g. non-homologous end joining [NHEJ], or single-strand annealing [SSA]) [34]. Both scenarios will ultimately trigger cell death via apoptosis. These discoveries have stimulated intensive research efforts focused on evaluating the therapeutic potential of PARP-1 inhibitors, principally for breast and ovarian cancers in BRCA-mutation carriers [35].

Expression levels of the PARP1 enzyme are significantly elevated in a variety of cancer types [36–42] compared with normal tissues, due to genomic stress, rapid proliferation, and abnormal metabolism. Furthermore, this enzyme has been found to have value as a prognostic indicator, particularly as the upregulation of PARP1 has been linked with reduced overall survival [38]. This is most notably the case in brain malignancies which frequently contain elevated levels of PARP1 while healthy brain tissue has extremely low basal expression of this enzyme [40, 41]. These observations strongly indicate that PARP1 is a valuable biomarker of DNA damage which could be detected by PET or SPECT imaging. Furthermore, as PARP1 is a well-established therapeutic target, radiotracers based on PARP-1 inhibitors could find application as companion diagnostics during therapy, since they could provide useful information during the drug development process regarding important aspects of in vivo behaviour, such as biodistribution, pharmacokinetics, and target engagement.

## Double-strand break repair mechanisms

**Fig. 3:**
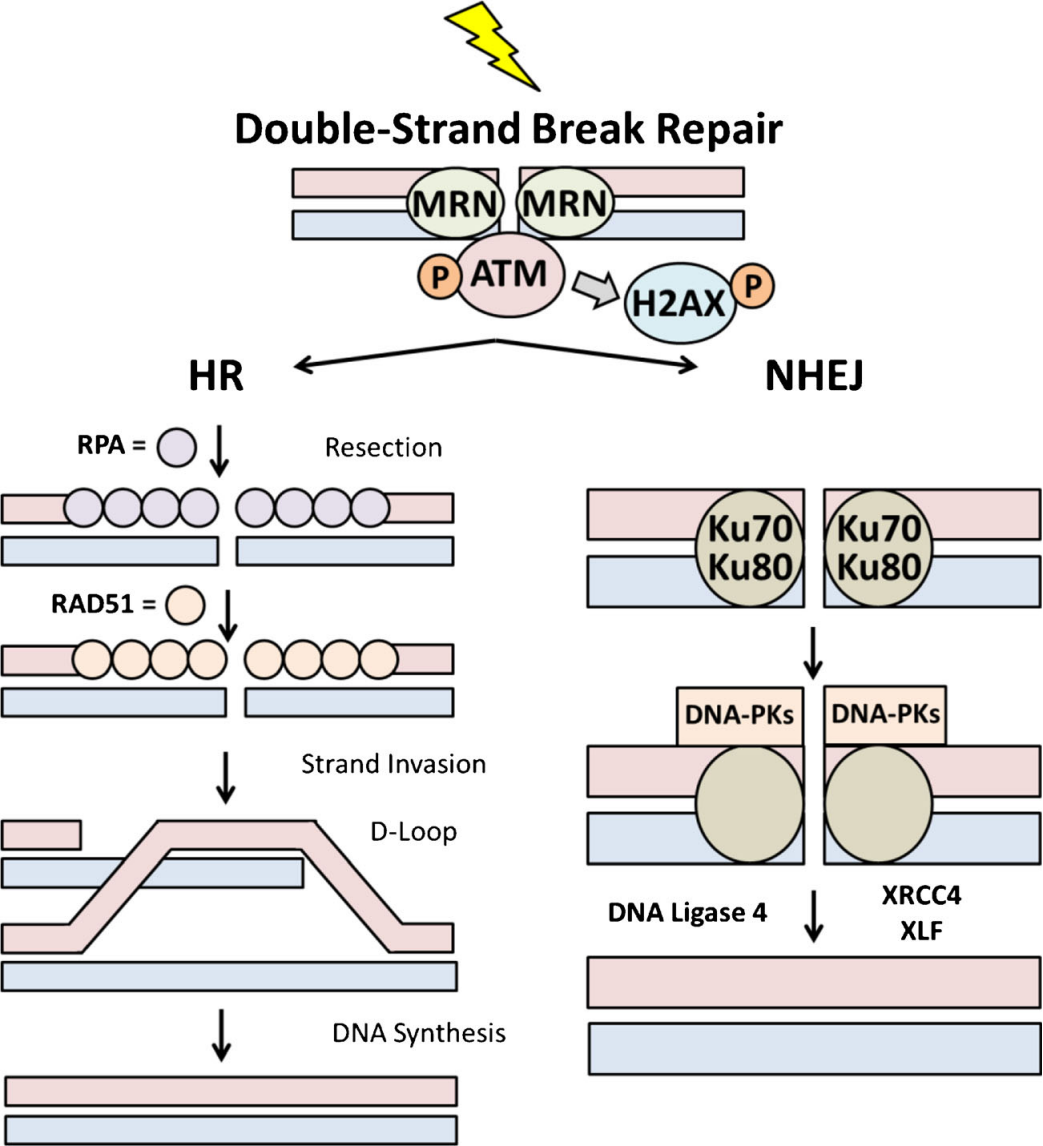
A simplified diagram of the principal steps in the repair of double strand breaks by homologous recombination (HR) and non-homologous end joining (NHEJ)

Double-strand breaks are the most harmful form of DNA damage as just a single occurrence can potentially result in chromosomal translocation or cell death [43]. The repair of DSBs is executed by two main pathways: HR and NHEJ (Fig. 3) [44].

HR is initiated when ataxia telangiectasia mutated (ATM) kinase protein binds to a DSB, whereupon it is activated and triggers the DNA damage response [45]. In the HR pathway, there are several protagonists, including the MRN complex (Mre11, Rad50, Nbs1), RPA, Rad51, and BRCA1/2. The MRN complex is responsible for the resection of 5’-3’ ends upon DSB recognition which are then coated with RPA [46]. Recombination is performed by Rad51, which replaces RPA in a BRCA1/2-dependant manner to assemble presynaptic Rad51 filaments [47]. A displacement loop (D-Loop) containing the novel heteroduplex DNA is then formed via DNA strand exchange between the target DNA and the Rad51 filament. Lastly, the broken 3’ end primes DNA synthesis using the duplex DNA as a template [48]. Several HR proteins are mutated in cancer, including BRCA1/2 in breast and ovarian cancer. These mutations can lead to inactivation of sub-pathways of HR, thereby driving other genetic effects responsible for the development of cancer.

NHEJ involves binding of the Ku70-Ku80 protein heterodimer to the DNA lesion, followed by the attachment of DNA-dependent protein kinase catalytic subunit (DNA-PKcs). The resulting DNA-PK holoenzyme binds and phosphorylates the protein Artemis, which cleaves the single-stand overhangs of DNA. Lastly, a complex of proteins, including DNA ligase 4 (LIG4), XRCC4 and XLF then complete the process by joining the DNA ends [49]. While NHEJ is less accurate than HR, it can be performed in the absence of undamaged sister chromatid DNA [50]. As with HR, NHEJ is important for genomic integrity since alterations of the Ku complex or LIG4 can cause genome rearrangements [51].

**Fig. 4:**
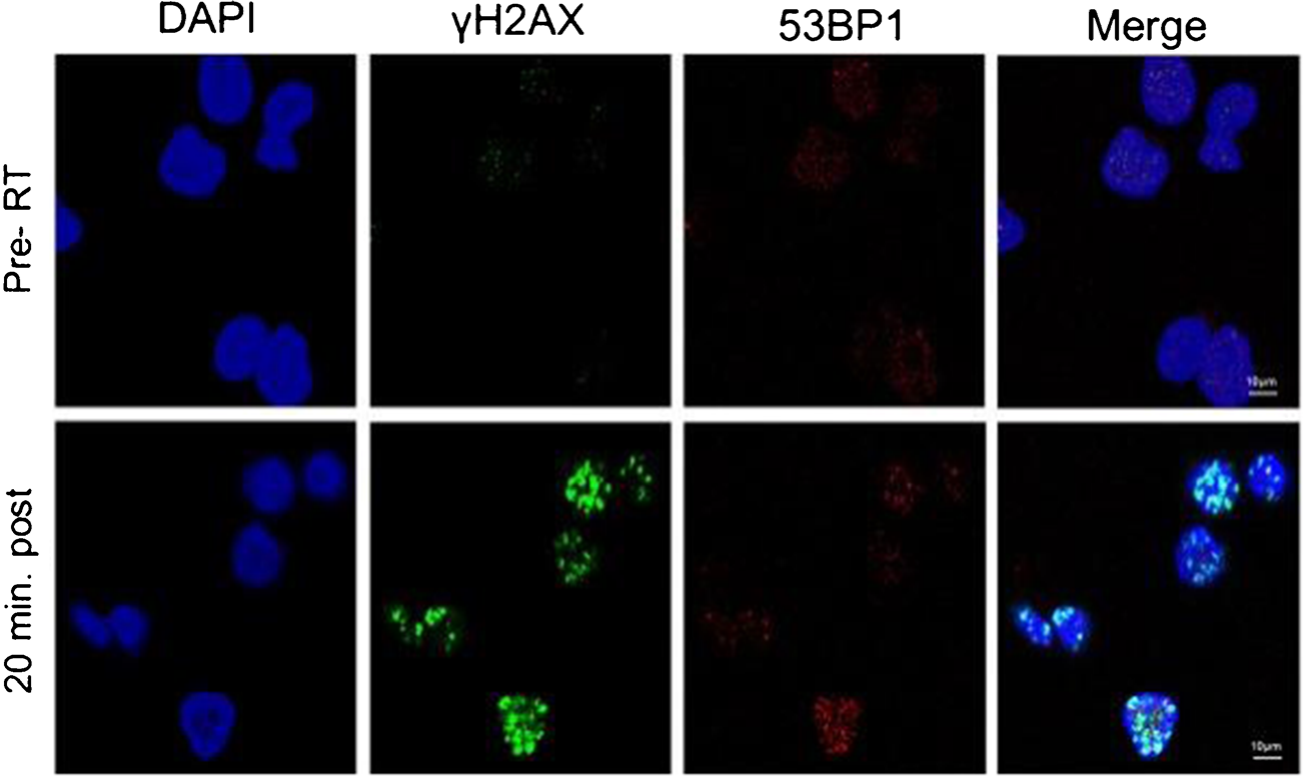
Immunostaining of fine-needle aspiration tumour specimens from a patient with non-Hodgkin’s lymphoma deposits reveals the appearance of γH2AX (green) and 53BP1 foci within the nucleus (DAPI, blue) 20 min following irradiation. Reproduced with permission from [54]

Shortly after a DSB event, the X isoform of the histone H2A is phosphorylated at the serine-139 position by members of the phosphoinositide 3-kinase (PI3K)-related protein kinase (PIKK) family such as ATM, ATR, and DNA-PKcs [52, 53]. The resulting protein, known as γH2AX, forms foci (Fig. 4 [54]) containing hundreds of copies (measuring up to 40 Mbp) [55] around each individual break site. Here, γH2AX is involved in the recruitment of most of the other DNA repair proteins discussed above, whereupon it promotes re-joining of DNA remnants [56, 57]. In addition, γH2AX regulates cell cycle checkpoints to ensure completion of DNA repair and chromatin structure around the affected site. After the damage is repaired, γH2AX is removed, restoring the affected parts of chromatin and preserving both genetic and epigenetic information [58]. The mechanism of removal is still not fully understood, but it has been proposed that it is mediated by dephosphorylation of γH2AX by phosphatases and through histone exchange in the chromatin [59, 60].

Phosphorylation of H2AX at serine-139 is abundant, rapid, and correlates well with each DSB, and consequently γH2AX has become the most commonly probed marker of DNA DSBs. γH2AX has added clinical value as it has been found to be expressed during the early development of most cancers. This includes bladder, breast, cervix, colon, lung, ovaries, pancreas, and skin cancers [57, 61, 62]. Furthermore, in clinical samples, high numbers of γH2AX foci have been correlated with poor outcomes [63]. Taken together, these properties render γH2AX an attractive target for quantitative, high sensitivity molecular imaging techniques.

## Methods for DNA damage detection: state-of the-art

One of the most well-established methods of probing DNA damage in vitro is pulsed-field gel electrophoresis (PFGE) [64]. While conventional gel electrophoresis techniques can resolve DNA fragments up to roughly 50 kb, the introduction of an alternating voltage gradient in PFGE is advantageous as it permits resolution of larger DNA fragments up to 10 Mb [65]. Another common electrophoresis-based method is the comet assay which, following separation of DNA fragments, leads to comet-like shapes which can be observed by fluorescence microscopy [66, 67]. The relative intensities of the head (undamaged DNA) and tail (damaged DNA) regions of each comet can be used to quantify SSBs and DSBs in individual eukaryotic cells; although this assay does not reveal the size of individual DNA fragments. Both PGFE and the comet assay have a common drawback as they rely on the extraction of damaged DNA from lysed cells prior to analysis.

The development of confocal immunofluorescence microscopy has since permitted visualisation of DDR proteins within the nuclear compartment of single cells and, in doing so, has helped to elucidate several key DNA repair mechanisms. For example, γH2AX and 53BP1 foci are routinely used to enumerate the extent of DSB repair following genotoxic stimuli [68, 69].

More recently, live cell imaging has provided valuable information on the kinetics of DNA repair in vitro [70]. Most live cell imaging experiments rely on transfection of the cell-of-interest with a gene coding for a chimeric fusion protein of the DDR protein-of-interest coupled to a fluorescent protein such as GFP, YFP, or mCherry. Examples include the use of a 53BP1-mCherry construct to study the kinetics of 53BP1 recruitment to DSB repair foci and their dissolution [71], and the use of Mdc1-, ATR-, and Chk1-GFP fusion proteins to probe the effects of ultraviolet laser radiation [72]. In 2009, Hilario et al. characterised the dynamics of Rad51 filaments and their assembly and disassembly to DNA by single molecule fluorescence microscopy [73]. This was one of the first demonstrations of a real-time study of Rad51 nucleoprotein filament formation, providing details on the rate of filament growth as well as the rate of DNA extension upon Rad51 association.

All of these studies have provided tremendous insight toward the spatiotemporal dynamics of DNA damage repair. The various genetic, biochemical, and molecular biological approaches that have been used to date have characterised in detail the different repair pathways involved. However, much more effort is still required to gain important insight such as how functional pathways are formed by coordination between different repair players, as well as the mechanisms involved in the interaction between these pathways and other cellular processes.

In an effort to extend some of the live cell DDR imaging techniques to an in vivo preclinical setting, Li et al. transfected H322 lung cancer cells with N- and C-terminal fragments of firefly luciferase genes fused with H2AX and MDC1, respectively [74]. Upon irradiation of the cells and formation of DSBs, MDC1 is recruited to phosphorylated H2AX in foci around the DSB, thereby bringing into close proximity both halves of the luciferase protein and allowing the formation of a visible light signal upon addition of luciferin. The same authors also showed that this approach is viable for use in mice bearing subcutaneous xenografts of transfected H322 cells [74]. An initial DDR response was observed in the first day after irradiation (6 Gy) of xenografts. A consecutive apoptotic response, reaching a maximum at 10 days post-irradiation, was also observed.

Later, a luciferase-based reporter was developed to non-invasively test ATM activity in cells and was found to undergo increased activation upon ATM inhibition in a dose-dependent manner, thus enabling the validation of ATM inhibitors in addition to quantifying ATM kinase activity [75]. This method could potentially allow the successful characterisation of ATM inhibitors used in therapeutic regimes, and was also evaluated for in vivo use. Both of these elegant methods allow repeated probing of aspects of the DDR, yet have the main disadvantage that they cannot be translated into clinical use, since transfection of the target cells is a necessity.

A much more advantageous prospect for translation to the clinic is the use of PET or SPECT imaging agents, particularly as they do not rely on modification of the target cell. These functional imaging techniques have excellent sensitivity in comparison to other clinical imaging modalities and are routinely used for in vivo tracking of biomolecular processes. The principal advantages that in vivo imaging of DDR can offer over conventional tissue biopsies are: (1) the ability to analyse larger tissue volumes compared with a small, potentially unrepresentative sample, (2) an improved insight into tumour heterogeneity, (3) the lack of need for an invasive operation to access the area of interest, which completely removes the risk of serious complications related to infection, haemorrhaging etc., and (4) the option to perform repeated imaging of the same area which would allow longitudinal assessment.

## Imaging PARP-1 with PET and SPECT

Because of the well-established role of PARP-1 as a mediator in the repair of DNA SSBs, it represents an attractive biomarker for PET and SPECT imaging. Consequently, over the last decade, there have been several attempts to develop radiolabelled imaging agents in order to permit visualisation of this DDR protein:

### [^11^C]-PARP-1 radiotracers

The first example of a PET radiotracer based on a PARP-1 inhibitor was reported by Tu et al. in 2005 [76]. In this case, a phenanthridinone derivative known as PJ34 was selected as it can block NAD^+^ from its binding site on the activated form of the PARP-1 enzyme. PJ34 was radiolabelled with carbon-11 via a base-catalysed reaction with [^11^C]methyl iodide and the resulting radiotracer, [^11^C]PJ34 (Table 1), was used in a rat model of type 1 diabetes to assess its ability to detect early stages of necrosis in pancreatic islets. Promisingly, [^11^C]PJ34 accumulated significantly more in necrotic pancreases compared with the pancreases of healthy rats at both 5 and 30 min after injection of the radiotracer.

**Table 1:** Pharmacological and (radio)chemical properties for a selection of radiolabelled PARP-1 inhibitors

Radiotracer	IC_50_ (nM)	*K*_i_ (nM)	Log P	Plasma protein binding	Plasma-free fraction (%)	Log D_7.4_	Blood half-life (min)	Specific activity (mCi/μmol)	Ref
[^11^C]PJ34	20	-	-	-	-	-	-	∼2,000	[76]
[^18^F]PARPi-FL	-	-	-	-	-	-	15.6	2.9±0.7 (manual) 9 (automated)	[77]
[^18^F]PARPi	2.8±1.1	-	logP_CHI_ = 2.15±0.41; logP_o/w_ = 1.76±0.18	-	63.9±12.6	-	α: 1.27 (85.51%); β: 31.14 (14.49%)	48	[78]
[^18^F]FluorThanatrace ([^18^F]FTT)	6.3	-	-	-	-	-	-	>2,200 [79], 5,500-18,000 [80]	[79, 80]
[^18^F]-BO	17.9±1.1	-	-	-	-	-	12.4	-	[81–83]
[^18^F]-4	-	200	-	-	-	1.4	-	-	[84]
[^123/124/131^I]-I2-PARPi	3.3[85] - 9±2[86]	-	Log P_CHI_ = 2.3[86]; Log P_o/w_ = 3[85]	96.2[85]	11.5±0.1[86]	-	17.1	145-210 ([^131^I]-I2-PARPi); 110–170 ([^124^I]-I2-PARPi; >19,000±300[^123^I]-I2-PARPi	[85, 86]

### [^18^F]-PARP-1 radiotracers

A handful of PARP inhibitors radiolabelled with fluorine-18 have also been evaluated and, in some cases, have shown good potential. Initial studies were focused on the preparation of radiofluorinated pirenzepine derivatives and related metabolites due to their ability to inhibit PARP-1 activity [84]. An early example, [^18^F]-4 (Table 1), reported by Riss et al. in 2009 exhibited a moderate binding affinity (*K*_i_) of 200 nM, good stability in human serum, and a logD_7.4_ value of 1.4, which would likely aid in penetration of cellular membranes although subsequent in vivo evaluation of this radiotracer has not been reported [84].

**Fig. 5:**
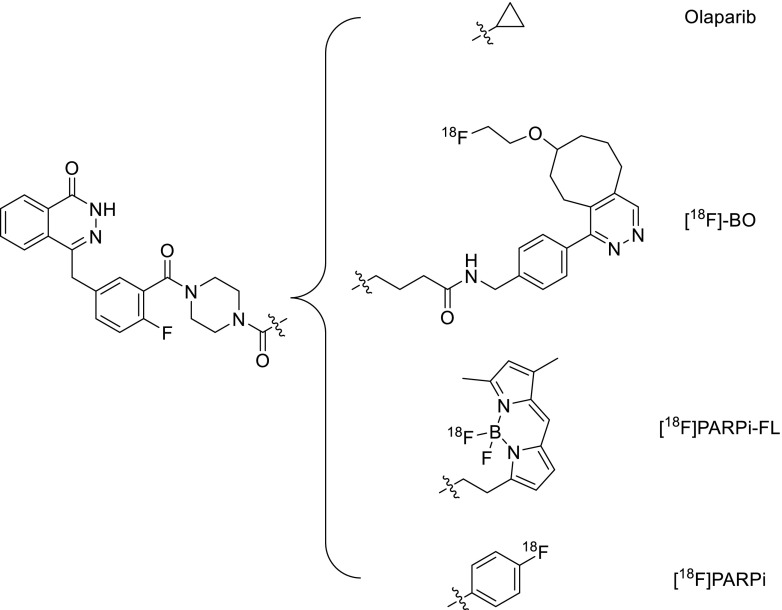
A selection of ^18^F-radiolabelled PARP-1 inhibitors derived from Olaparib

**Fig. 6:**
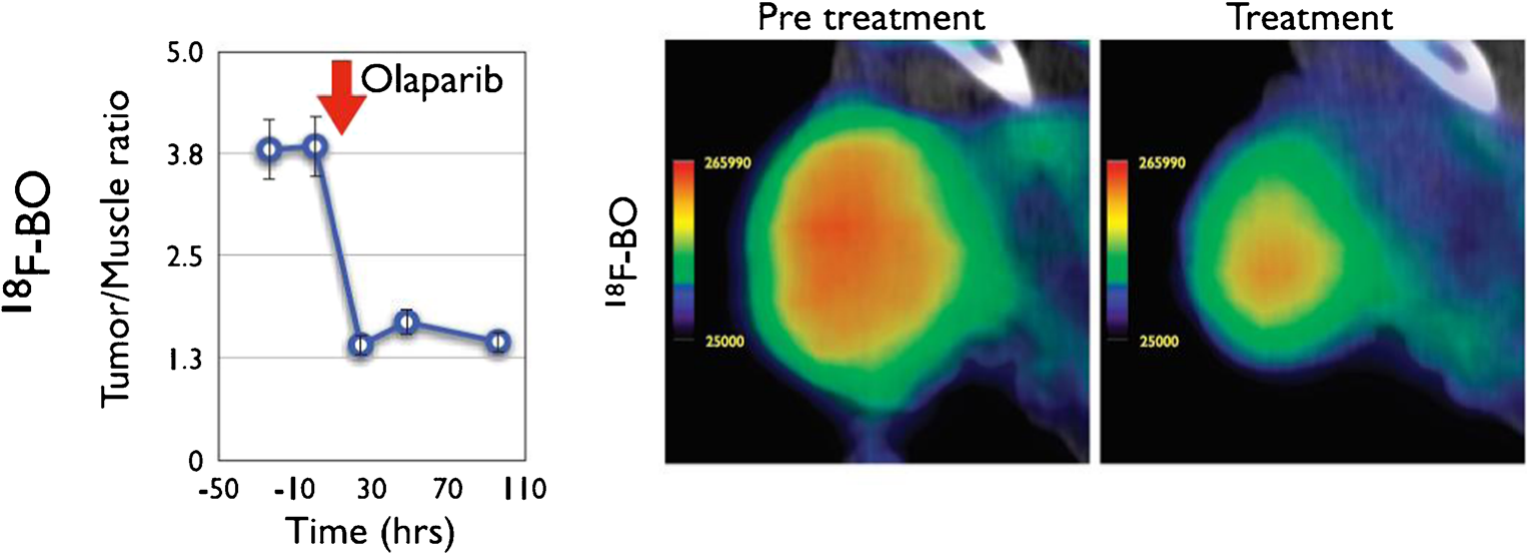
Reiner et al. demonstrated that measuring response to Olaparib treatment is possible using [^18^F]-BO [83]. Left: In mice bearing A2780 tumour xenografts, tumour-to-muscle contrast ratios markedly reduce following administration of Olaparib. Right: Representative PET/CT images pre- and post-Olaparib administration. Reproduced with permission from [83]

Weissleder and colleagues subsequently reported an ^18^F-radiolabeled derivative of the much-studied PARP inhibitor Olaparib, [^18^F]-BO (Fig. 5, Table 1), which was synthesised via a [4+2] inverse-electron-demand Diels-Alder cycloaddition reaction between a tetrazine-modified Olaparib derivative and an ^18^F prosthetic group based on *trans*-cyclooctene [81–83]. The half maximal inhibitory concentration of the resulting compound (IC_50_ = 17.9±1.1 nM) was only moderately reduced relative to unmodified Olaparib (IC_50_ = 5 nM) [87], which indicates that minor chemical modifications to the piperazine moiety of Olaparib are only minimally disruptive to PARP-1 binding. In addition to showing PARP-1-mediated cellular uptake in in vitro assays, [^18^F]-BO was also shown in PET experiments to accumulate specifically in PARP-1 overexpressing MDA-MB-468 breast cancer xenografts in mice [82]. Reiner et al. later demonstrated that [^18^F]-BO could accurately measure the extent of PARP-1 expression in a variety of xenograft tumour models in mice and showed that uptake of [^18^F]-BO in ovarian (A2780) xenograft tumours was markedly reduced after administration of Olaparib (Fig. 6) [83]. These compelling findings indicate the potential of this radiotracer to be used as a companion diagnostic for measuring therapeutic drug inhibition in an in vivo setting.

In an effort to create a dual-modality PARP imaging agent, Carlucci et al. developed an Olaparib derivative ([^18^F]PARPi-FL, Fig. 5, Table 1) containing both BODIPY-FL dye and a fluorine-18 atom which was introduced via an ^18^F/^19^F trans-fluorination exchange reaction [77]. While relatively facile, this method of radiolabeling led to low specific activities (2.9±0.7 or 9 mCi/μmol using manual and automated synthesis methods, respectively) compared to the other PARP inhibitors discussed herein. Furthermore, [^18^F]PARPi-FL was subject to rapid metabolic defluorination, which resulted in high bone uptake (∼10-15%ID/g). This imaging agent was capable, however, of distinguishing U87 glioblastoma xenografts (0.78±0.1%ID/g at 90 min post injection) in small animal PET imaging and ex vivo biodistribution experiments, which was effectively blocked (0.15±0.06%ID/g) with an excess of Olaparib.

**Fig. 7:**
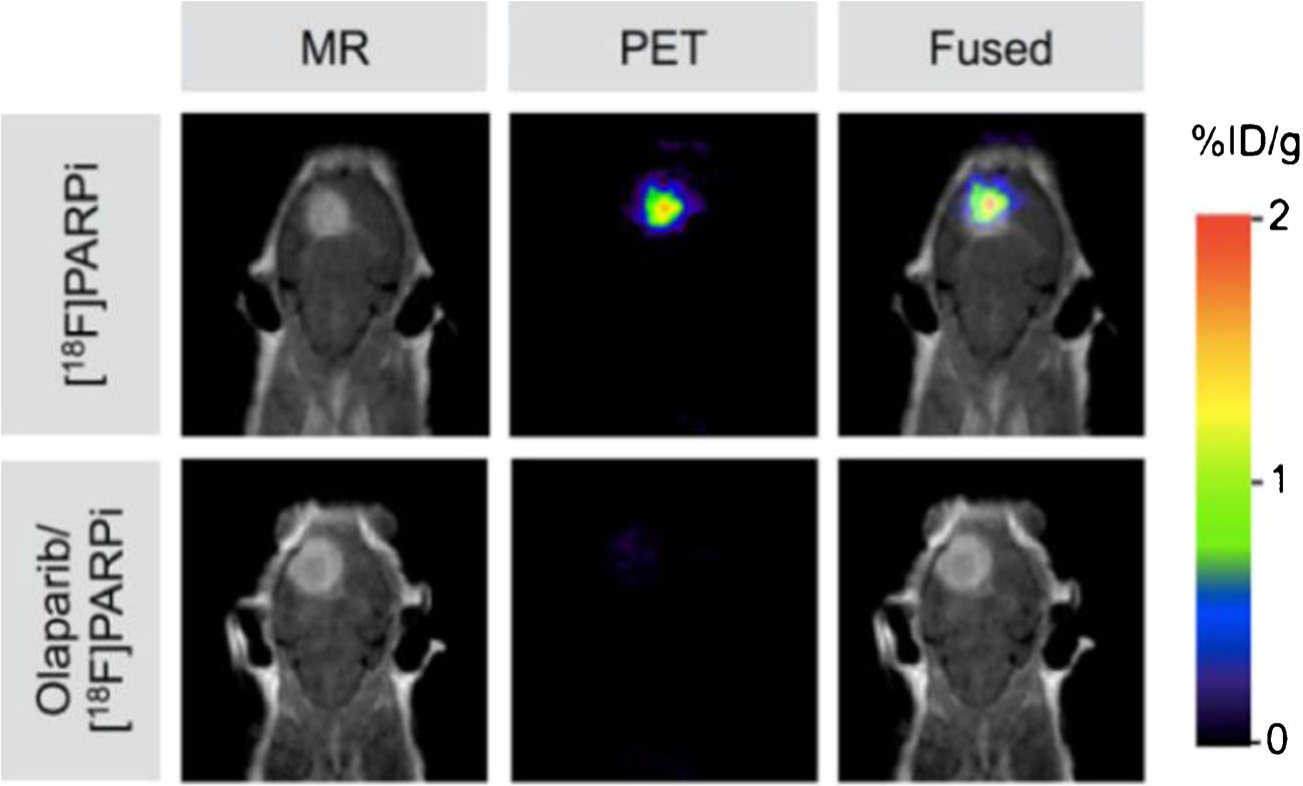
Top: In orthotopic glioblastoma-bearing mice, PET/MRI images showed pronounced uptake of [^18^F]PARPi at 2 h post-injection. Bottom: Pre-injection with a 500-fold excess of Olaparib effectively reduced tumour uptake of [^18^F]PARPi, providing evidence of specificity of the imaging agent for PARP-1. Reproduced with permission from [78]

While [^18^F]-BO and [^18^F]PARPi-FL each contain bulky chemical substituents, a more recent study has resulted in an [^18^F]-radiolabeled compound which is structurally more consistent with the parent molecule, Olaparib [78]. This radiotracer, [^18^F]PARPi (Fig. 5, Table 1), has an attractive IC_50_ value of 2.83 nM and, in contrast to [^18^F]PARPi-FL, the aromatic carbon-[^18^F]fluorine bond exhibited high stability, remaining largely intact in human serum samples over 4 h. In ex vivo biodistribution experiments performed at 2 h post injection, this radiotracer achieved uptake values of 1.82±0.21%ID/g in subcutaneous U87 xenograft tumours, which was proven to be mediated by PARP-1. In an orthotopic glioblastoma model, [^18^F]PARPi was also shown to selectively accumulate in PARP-1-expressing U251-MG tumours (Fig. 7), indicating the ability of this radiotracer to pass through the blood–brain barrier. In a similar manner to the other radiotracers derived from Olaparib discussed herein, the majority of [^18^F]PARPi excretion occurs via the hepatobiliary clearance pathway.

**Fig. 8:**
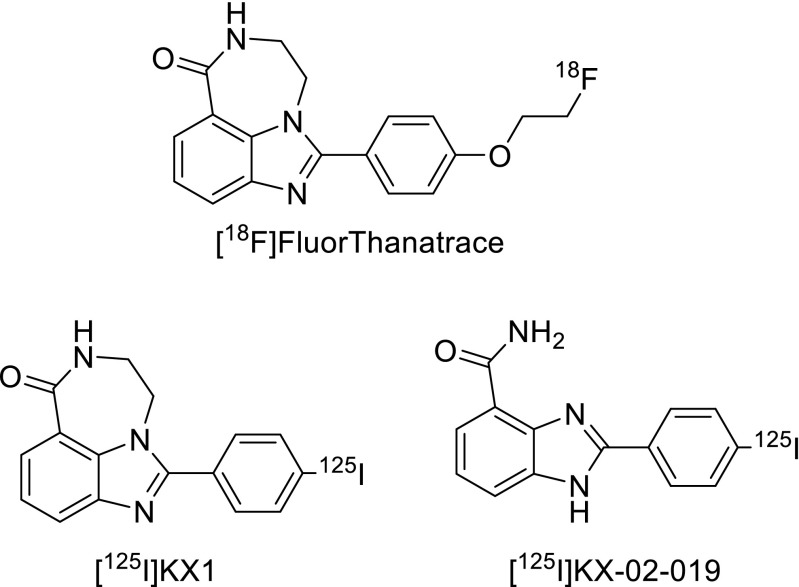
A selection of radiolabelled PARP-1 inhibitors based on benzimidazole derivatives

In 2014, Zhou et al. showed that radiofluorinated derivatives of the benzimidazole NU1085 and its structural near-relative AG014361 also had high inhibitory potency against the PARP-1 enzyme [80]. One of these structures, known as [^18^F]FluorThanatrace ([^18^F]FTT; Fig. 8, Table 1), was prepared in considerably higher specific activities compared to the other PARP-1 radiotracers discussed herein (5,500-18,000 mCi/μmol) and was also found to have a reasonable IC_50_ value of 6.3±1.3 nM. Small animal PET/CT experiments comparing MDA-MB-231 (low PARP-1 expressing) and MDA-MB-468 (high PARP-1 expressing) xenograft tumours revealed PARP-1 mediated tumour uptake which could be blocked following pre-injection of either Olaparib or [^19^F]FTT. Edmonds et al. also recently used this compound, albeit at a significantly lower specific activity (<2,200 mCi/μmol), and showed that uptake of this radiotracer in a variety of xenograft tumour models in mice could be correlated with intrinsic PARP-1 expression levels. This study also provided compelling evidence that uptake of [^18^F]FTT is mediated solely by PARP-1, which was concluded after in vitro uptake experiments in PARP-2 knock-out cells revealed specific binding at levels comparable to wild-type cells [79].

### Radioiodinated PARP-1 radiotracers

There have been several recent examples of PARP-1 inhibitors labeled with radioisotopes of iodine for both PET and SPECT imaging applications [85, 86]. Two of these reports describe the development of multiple radioiodinated Olaparib derivatives; however, each focuses on the evaluation of a single structurally identical compound, [^123/124/131^I]-I2-PARPi (Table 1). The Reiner group demonstrated the feasibility of using this compound to detect glioblastoma by targeting orthotopic U251 MG xenografts in mice [86]. SPECT/CT and PET/CT studies involving [^131^I]-I2-PARPi and [^124^I]-I2-PARPi, respectively, enabled visualisation of PARP-expressing tumour tissue that could be readily delineated from normal brain tissue. In particular, [^124^I]-I2-PARPi yielded attractive tumour-to-brain and tumour-to-muscle ratios of 40.0±6.3 and 13.7±4.1, respectively, at 2 h post-injection. Zmuda et al. also demonstrated in a subcutaneous U87MG-Luc2 glioblastoma xenograft model (WHO grade IV) the ability of [^123^I]-I2-PARPi to be retained within tumour tissue and correlated this uptake to the expression of PARP and the proliferative marker Ki67 [85]. In this case, a peak tumour-to-muscle ratio of 5.61±1.99 was achieved at 2 h post injection.

Mach and colleagues have reported two radioiodinated benzimidazole derivatives, [^125^I]KX1 [88] and [^125^I]KX-02-019 [89] (Fig. 8), which bear close structural resemblance to FluorThanatrace. The ability of [^125^I]KX1 to measure PARP-1 expression in vivo was tested in mice bearing subcutaneous HCC1937 (high PARP-1) and MDA-MB-231 (low PARP-1) human breast cancer xenografts [88]. At 2 h post injection, significantly higher uptake of [^125^I]KX1 was observed in HCC1937 tumours (reaching approximately 5%ID/g); however, no blocking effect was observed following administration of olaparib suggesting a lack of specificity. It was postulated that differences in the pharmacokinetic profiles of these two agents could prevent a blocking effect, although no additional attempts at blocking with unlabelled KX1 were reported. Autoradiography analysis of HCC1937 tumour tissue at 2 h post injection did however reveal a reduction of signal following Olaparib treatment compared to non-treated mice. It could, therefore, be envisaged that this agent, and analogous agents containing iodine-123/124/131, could serve as companion diagnostic agents during therapy and may assist in patient stratification.

## Imaging γH2AX with PET and SPECT

The ability to monitor γH2AX expression in vivo may help to detect certain cancers earlier in their development compared with existing diagnostic methods, facilitating timelier intervention and improved survival. Furthermore, it would also allow indirect monitoring of the DSBs caused by radiotherapy and some chemotherapeutic agents, thus permitting rapid determination of therapeutic efficacy. Consequently, a concerted effort is now underway to develop a non-invasive means of quantifying γH2AX expression levels in vivo using both PET and SPECT imaging techniques.

As an imaging biomarker, γH2AX has several advantages compared with other DDR proteins. For example, H2AX can be phosphorylated throughout the cell cycle, whereas 53BP1, MRE11, and NBS1 are dissociated from DNA damage foci during mitosis [56, 90, 91]. Furthermore, 53BP1 is known to form DNA repair foci via translocation during, which its expression levels do not change dramatically. While PET and SPECT imaging are very sensitive imaging techniques, they cannot distinguish between the various intracellular locations of proteins such as 53BP1. In stark contrast, γH2AX is a new species which is induced by phosphorylation following DSB formation. This on/off switch-like behaviour renders the γH2AX a much more attractive imaging target, i.e. under physiological conditions, cells express little to no yH2AX, whereas upon DSB formation, γH2AX is formed very rapidly.

### γH2AX radiotracer development

While anti-γH2AX antibodies are now used routinely in ex vivo assays to quantify the number of γH2AX foci within permeabilised cell populations, the translation of such antibody-based imaging agents into an in vivo setting requires the addition of the cell penetrating peptide (CPP) [92, 93] named “TAT”, which is derived from the transactivator of transcription protein of the HIV-1 virus [94–98]. This arginine-rich peptide has been shown to promote the cellular internalisation of antibodies and a variety of other species, including peptides [99], nanoparticles [100], and liposomes [101]. The precise mechanism(s) of internalisation have been the focus of several studies, many of which have provided strong evidence that electrostatic interaction of the positively charged CPP with negatively charged heparin sulfate proteoglycans on the periphery of the cell membrane plays an important role in promoting internalisation via endocytosis [102, 103]. However, it is worth noting that none of these studies have been able to inhibit completely this process, which suggests that other mechanisms, including energy-independent direct translocation, could also be a contributing factor to this phenomenon. Of the endocytotic pathways that could be responsible, virtually all known possibilities have been implicated, including macropinocytosis [104], clathrin- [105], and caveolin-mediated endocytosis [106]. It is of course possible, if not likely, that more than one mechanism of TAT-internalisation exists and therefore these studies are not necessarily contradictory. The TAT peptide is also known to contain a nuclear localisation sequence (NLS), which, through binding to importins [107], is further trafficked into the nuclear compartment of the cell whereupon the anti-γH2AX antibody can bind to its target.

It is important to note that the CPP does not impart specificity for any particular cancer biomarker and, therefore, it is crucial that the whole antibody construct is able to be externalised so that it can have further opportunity to reach its target. An externalisation mechanism for the TAT protein has been ascertained in a series of elegant experiments which implicate binding to phosphatidylinositol-(4,5)-bisphosphate on the inner leaflet of the cell membrane [108]. The net result of these concurrent cellular import/export mechanisms is that cells in a more active state of DNA damage repair will retain the anti-γH2AX-TAT construct for longer time periods compared with healthy cells with low basal expression levels of γH2AX.

The addition of TAT to the antibody is typically achieved using conventional EDC/sulfo-NHS coupling reagents which promote the formation of an amide bond between the terminal primary amine of TAT and any accessible carboxylic acid residue on the antibody. Alternative bioconjugation methods involving modification of carbohydrate groups on the Fc chain have also been employed successfully [109].

### Examples of γH2AX imaging with PET and SPECT

**Fig. 9:**
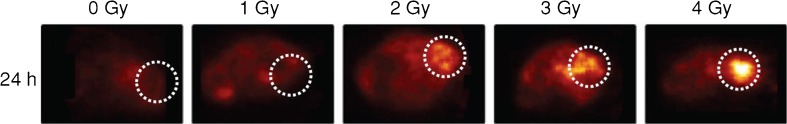
Cornelissen et al. showed that uptake of ^111^In-anti-γH2AX-TAT in MDA-MB-468 breast cancer tumours increased following irradiation in a dose-dependent manner. Reproduced with permission from [110]

Our first report of a TAT-modified anti-γH2AX antibody emerged in 2011 [110]. Here, the construct was labelled with either a fluorophore or the SPECT radioisotope ^111^In. When labelled with the fluorophore Alexa Fluor® 488 (λ_ex/em_: 495/519 nm) this construct was shown in in vitro experiments to gradually internalise over the course of 23 h into MDA-MB-468 human breast cancer cells which had been exposed to DNA damaging radiation (4 Gy). Promisingly, this compound formed discrete foci within the nuclear compartment which strongly co-localised with staining for γH2AX. The radioactive ^111^In-anti-γH2AX-TAT compound was also found to be retained significantly longer within irradiated cells compared with a series of experimental controls. The in vivo evaluation of ^111^In-anti-γH2AX-TAT showed an ability to track DNA damage using a MDA-MB-468 xenograft tumour model in mice (Fig. 9). Here, DNA damage within the tumours was induced by either irradiation or via administration of bleomycin. In both cases, higher uptake of ^111^In-anti-γH2AX-TAT was observed in the tumours of mice that had received therapy. Taken together, these experiments provided the first compelling evidence of the feasibility of using CPP-modified antibody constructs to image intracellular DDR targets.

**Fig. 10:**
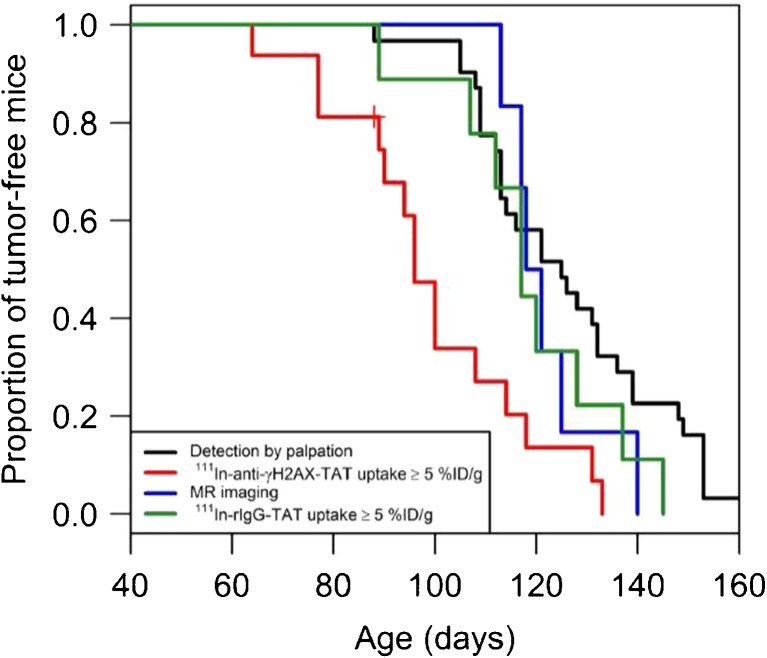
A Kaplan-Meier plot revealing that precancerous lesions and tumours in BALB-neuT mice could be positively identified by ^111^In-anti-γH2AX-TAT SPECT imaging at a younger age compared with palpation or DCE MR imaging

The ability of ^111^In-anti-γH2AX-TAT to image non-invasively DDR during oncogenesis has since been evaluated in a genetically engineered mouse model of HER2/neu-overexpression driven breast cancer [111]. This model results in the development of multiple palpable carcinomas in the mammary fat pads when mice reach 130 days old. In a longitudinal study, SPECT images were acquired on a weekly basis at 24 h post-injection of ^111^In-anti-γH2AX-TAT. In SPECT images acquired from mice between 76–110 days old, uptake of the radiotracer in the mammary fat pads was markedly higher compared with a non-specific isotype-matched antibody which was modified in an identical manner. Immunohistochemical analysis of resected mammary fats pads showed that the number of γH2AX foci per cell reached a peak within this age range and was significantly greater compared with mice <76 or >106 days old. Encouragingly, it was found that the median time to the detection of positive tissue with SPECT imaging (96 days) was much earlier compared with the detection of lesions >150 μm by DCE-MRI (120 days) or by palpation (131 days) (Fig. 10).

While advances in SPECT technology (specifically relating to improvements to collimators, quantitation, and reconstruction software) are leading to a revival in this modalities appeal, PET/CT imaging has gained acceptance as the standard of care in the management of cancer. This is due in part to the high resolution and sensitivity that clinical PET has so far offered compared with SPECT, and the ability during processing of PET images to accurately correct for signal attenuation. These important advantages have resulted in higher quality images from which more meaningful data can be extracted. Consequently, a PET radiotracer based on the anti-γH2AX-TAT antibody construct has been developed containing the radiometal zirconium-89.

The performance of ^89^Zr-anti-γH2AX-TAT has largely shown consistency with its indium-111 radiolabelled analogue [112]. In cells exposed to DNA damaging radiation (4 Gy), ^89^Zr-anti-γH2AX-TAT exhibits considerably (eightfold) longer retention compared with control experiments involving non-irradiated cells or a non-specific IgG. Furthermore, in subcutaneous MDA-MB-468 xenograft tumours in mice, higher uptake of ^89^Zr-anti-γH2AX-TAT (0.5 MBq, 5 μg) was found following exposure to radiation (10 Gy) compared with experimental controls (12.1 ± 1.6%ID/g, *P* < 0.001).

Radiotracers based on anti-γH2AX-TAT constructs have shown promise in a variety of preclinical models based on early detection and therapy evaluation. However, prior to clinical translation, some important issues require consideration. Firstly, it will be important to integrate a humanised version of the anti-γH2AX antibody (the antibody used in these studies is raised in rabbit) in order to prevent the invocation of an immune response. Secondly, as non-specific tumour uptake (resulting from the enhanced permeability and retention [EPR] effect [113]) is responsible for a substantial contribution to overall tumour uptake, it will be desirable to amplify γH2AX-mediated contrast. We hypothesise that this could be achieved by improving delivery of the construct to tumours by, for example, attachment of tumour-targeting peptides [114]. This may also be achieved by reducing non-specific uptake resulting from EPR by either using smaller antibody fragments (minibodies, diabodies, etc.) [115] or by adopting a pretargeted imaging approach [116].

Lastly, it is worth noting that γH2AX is a secondary marker of DNA DSBs and its expression or foci number is not a direct 1:1 measure of DSBs. This leads to difficulty in quantifying precise numbers of DSBs that are being visualised in vivo, especially when considering the relatively slow pharmacokinetic profile of antibody-based imaging agents. Therefore, it is important that the biology of yH2AX is taken into account when interpreting images and image quantitation. At present, no in vivo imaging modality exists that is sufficiently sensitive to image the DSBs directly.

## Conclusions

Our understanding of the cellular processes that are invoked in response to DNA damage has improved considerably over recent years. Advancements in this area have revealed attractive biomarkers, which could be used to improve upon existing methods for non-invasive early cancer detection and therapy evaluation. With these aims in mind, a range of PET/SPECT imaging agents are currently under development and, in some cases, are poised for evaluation in clinical settings. So far, these imaging agents have mostly consisted of small molecule inhibitors of the PARP-1 enzyme or antibody-based constructs targeting yH2AX.

Most of the reports described herein have demonstrated the ability to measure PARP-1 or yH2AX expression levels in preclinical in vivo experiments and some studies have been able to detect changes in expression following chemo- or radiotherapy. While these findings are very promising, it should be noted that uptake of these agents within tumours is generally low, particularly in comparison to what can often be achieved through targeting of cancer biomarkers situated on the cell-surface. This can be attributed to a variety of factors, including the transient nature of DDR proteins, and the inefficient internalisation/nuclear translocation of these agents. While the first of these challenges is an inherent and unavoidable obstacle in this endeavour, the latter is an area which can conceivably be improved upon through advances in the understanding of the various internalisation mechanisms and by improved chemical design.

There is also a need to identify which of the multitude of proteins involved in the DDR process represent the most valuable targets. This complex task will involve measuring intranuclear concentrations of target proteins at various stages during oncogenesis (as well as during cancer therapy), and determining relative abundances in cancerous versus healthy tissues. The duration of the targets’ existence is also an important parameter, particularly if it will be used to assess DDR activation following radiotherapy where expression levels of many biomarkers, including PARP-1 and γH2AX, disappear within days.

Of equal importance is the need to develop improved PET and SPECT imaging agents which can bind to these targets with high affinity and specificity, while also possessing the necessary chemical properties that would promote efficient cellular internalisation. Certainly, this will be aided by recent advances in radiofluorination chemistry which allow far greater freedom in the design of novel PET radiotracers compared to what has previously been possible with conventional radiosynthetic approaches.

More broadly, non-invasive imaging of intracellular targets is an important research endeavour with implications which extend beyond imaging the DNA damage response. This ability also opens the door to a multitude of other intracellular biomarkers with relevancy to additional aspects of cancer biology.
